# The effects of awareness of the perturbation during motor adaptation on hand localization

**DOI:** 10.1371/journal.pone.0220884

**Published:** 2019-08-09

**Authors:** Shanaathanan Modchalingam, Chad Michael Vachon, Bernard Marius ‘t Hart, Denise Y. P. Henriques

**Affiliations:** 1 Centre for Vision Research, York University, Toronto, Ontario, Canada; 2 School of Kinesiology and Health Science, York University, Toronto, Ontario, Canada; 3 Department of Psychology, York University, Toronto, Ontario, Canada; West Virginia University, UNITED STATES

## Abstract

Awareness of task demands is often used during rehabilitation and sports training by providing instructions which appears to accelerate learning and improve performance through explicit motor learning. However, the effects of awareness of perturbations on the changes in estimates of hand position resulting from motor learning are not well understood. In this study, people adapted their reaches to a visuomotor rotation while either receiving instructions on the nature of the perturbation, experiencing a large rotation, or both to generate awareness of the perturbation and increase the contribution of explicit learning. We found that instructions and/or larger rotations allowed people to activate or deactivate part of the learned strategy at will and elicited explicit changes in open-loop reaches, while a small rotation without instructions did not. However, these differences in awareness, and even manipulations of awareness and perturbation size, did not appear to affect learning-induced changes in hand-localization estimates. This was true when estimates of the adapted hand location reflected changes in proprioception, produced when the hand was displaced by a robot, and also when hand location estimates were based on efferent-based predictions of self-generated hand movements. In other words, visuomotor adaptation led to significant shifts in predicted and perceived hand location that were not modulated by either instruction or perturbation size. Our results indicate that not all outcomes of motor learning benefit from an explicit awareness of the task. Particularly, proprioceptive recalibration and the updating of predicted sensory consequences appear to be largely implicit. (data: https://doi.org/10.17605/osf.io/mx5u2, preprint: https://doi.org/10.31234/osf.io/y53c2)

## Introduction

When performing movements, it is crucial to know the location of your limbs in space. Such estimates of limb position are based on two general sources. One is afferent based, relying on sensory feedback, primarily vision and proprioception, and the second is efferent based, simulating or predicting the consequences of motor commands [1]. Proprioceptive and predictive estimates of your limb positions should, and have been shown to, change as a consequence of motor learning [2–4]. Motor learning can also be partitioned into two separate processes; unaware implicit learning and aware explicit learning [5–9]. Although changes in estimates of hand position and the multiple processes of motor learning have been studied separately, it is not yet clear how they interact. Specifically, we do not yet know whether proprioceptive and predictive changes in hand estimates are primarily implicit, or if can they be influenced by explicit awareness during learning.

In this study, in order to test if explicit processes affect these different estimates of hand position, we test whether awareness of perturbations when reaching with a visuomotor rotation influences resulting changes in estimates of hand position. We manipulate awareness in two ways: by providing some participants with instructions on the nature of the perturbation, and by having some participants experience a large, conspicuous perturbation. We then test how differences in awareness, and the resulting increase in explicit learning, affect changes in estimates of the position of the adapted hand.

Adaptation involves both implicit, and explicit conscious processes. Typically, adaptation to small perturbations is thought to be implicit, where there is a change in an internal forward model due to sensory prediction errors brought about by the perturbation [10,11]. The implicit processes associated with motor adaptation may be gleaned by measuring the reach aftereffects; the continued change in performance even in the absence of the perturbation [12–14]. However, it has been suggested that people also use cognitive strategies to aid in the adaptation process, especially during early adaptation [8,9,15]. Likewise, this explicit component of adaptation can be elicited and further increased with prior instruction [5,7,15–17] and with larger rotations [5–7,18]. Here we attempt to evoke awareness during the learning process by providing a large rotation (60° as opposed to a small 30° rotation), or by instructing participants on the nature of the rotation by providing a detailed explanation and a strategy to counter the perturbation.

When the perturbation involves altered visual feedback of the hand, our estimates of hand position based on visual inputs are decoupled from those based on proprioceptive input. After training with this visual discrepancy, participants’ reports of their unseen hand location shift; usually on the order of 20% of the visual distortion [2,4,19–21]. This proprioceptive recalibration is robust across different variations in the perturbations [2,4,22] and emerges quite quickly [21]. While not directly tested, participants appear to be unaware of these small shifts of estimate of hand location, and this in turn may contribute to the implicit component of adaptation.

Additionally, when we produce movements, such as reaches, we generate predictions of the consequences of the initiated motor command based on a copy of the motor command. Introducing perturbations causes a consistent discrepancy between these predicted consequences of a movement and the actual sensory consequences, or feedback, during the movement. Models of motor learning suggest that this discrepancy is what drives implicit motor adaptation [23,24]. This efferent-based change in performance, which aims minimize this sensory prediction error, can be partly captured by measuring changes in estimates of the location of the hand following volitional movements [1,3,25].

Given that these modifications of both movement and perception of the hand are largely implicit, it is unknown whether making the perturbation explicit should result in reduced recalibration of these estimates. When people are aware of the rotation and what they are doing to counter it, it is almost certain that the visual feedback does not represent the true location of their hand. Thus, there is no need to update either hand-localization signal. However, it is unknown whether and to what degree this reduction may occur. In this study, we test whether proprioceptive recalibration and updating of the predicted consequences of movements are minimized by awareness of a perturbation during visuomotor adaptation.

## Methods

### Participants

Eighty-four right-handed participants from York University took part in the experiment. All participants gave prior, written, informed consent and participation was voluntary. The procedures used in this study were approved by the York Human Participants Review Sub-committee. All participants reported having normal or corrected-to-normal vision.

Forty-one of the participants adapted to a 30° visuomotor rotation, and the other forty-three participants to a 60° rotation. To ensure awareness during training, these two groups were further split by giving explicit instructions on the nature of the perturbation, and strategies to counter the rotation, to half of the participants who experienced each rotation size. Thus, there were four groups: non-instructed 30° (n = 20, 14 female), instructed 30° (n = 21, 13 female), non-instructed 60° (n = 20, 14 female) and instructed 60° (n = 24, 18 female). We visually inspected all tasks to ensure participants followed their given instructions. We excluded 8 participants who failed to complete the tasks as instructed (not included in the counts above).

### General set-up

**Fig 1:**
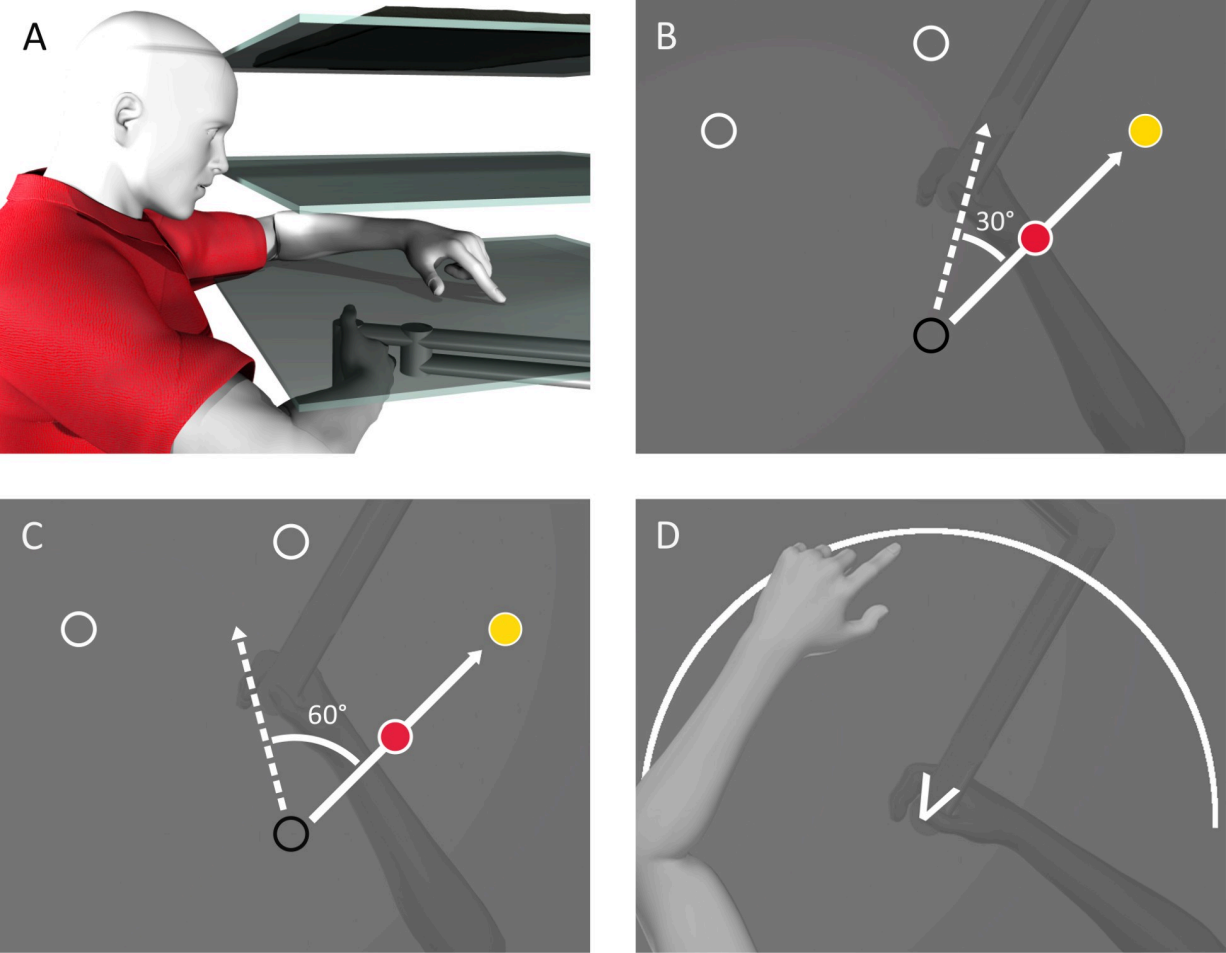
Experimental setup and procedure. A) Participants gripped a robot manipulandum located below a touchscreen (bottom surface) while looking at a reflective screen (middle surface). The reflected visual stimuli were projected from a monitor (top surface) located above the reflective screen. B and C) During ‘Reach to Target’ tasks in the ‘Rotated’ session, the position of a cursor representing the hand (red and blue circle) was rotated 30° (B) or 60° (C) CW during rotated training tasks. Participants attempted to move the cursor to one of the yellow targets as quickly and as straight as possible. D) During localization tasks, participants used their visible left hand to indicate where they crossed a visible arc with their unseen right hand. For participants in the 60° rotation groups, a V-shaped wedge served as an indicator as to which part of the workspace to move their unseen hand.

Participants were seated on a height-adjustable chair in front of an apparatus (Fig 1A) which included a downward facing computer screen (Samsung 510 N, 60 Hz) located 28cm above a 2-joint robot manipulandum (Interactive Motion Technologies Inc., Cambridge, MA, USA) with an attached vertical handle. A semi-reflective surface, located 14cm above the robot manipulandum, was used to reflect images displayed on the computer screen, allowing them to be projected on the same horizontal plane as the manipulandum. A touchscreen (Keytec Inc., Garland, TX, USA; resolution of 4096x4096 pixels), which participants sometimes used to localize their unseen right hand, was located 2cm above the manipulandum. The chair’s height was adjusted until the participant could manipulate and reach with the robot handle comfortably while viewing the entire display on the reflective surface. Participants were asked to grip the handle on the manipulandum with their right hand. A thick black cloth was draped over each participant’s right shoulder and arm which occluded their view of their right arm. The experiment was performed in the dark to limit peripheral vision of their right arm. Each participant’s left hand was illuminated by small lamps. All visual stimuli were presented via the reflective surface using the downward facing screen.

### Procedure

**Fig 2:**
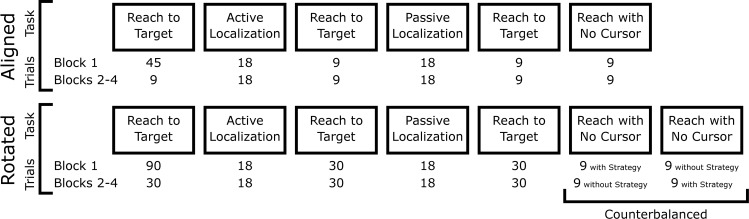
Sequence of tasks. All four groups followed the same sequence of tasks. Participants completed four blocks of the aligned session of the experiment and took a mandatory 10-minute break before moving onto the rotated session. The “Reach to Target” tasks in the Rotated session either included a 30° or a 60° CW visuomotor rotation, depending which group the participant belonged to.

All participants completed ‘Reach to Target’ tasks followed by ‘Localization’ and ‘Reach with No Cursor’ tasks (Fig 2 and described in detail below). During ‘Reach to Target’ tasks in the ‘Aligned’ session of the experiment (top row of Fig 2), the location of a cursor representing the participant’s unseen hand was aligned with the real position of the unseen right hand. These tasks in the ‘Aligned’ session served both to familiarize participants with the tasks and to measure baseline data. After ‘Localization’ and ‘Reach with No Cursor’ tasks, participants completed top-up ‘Reach to Target’ trials. After completing blocks 1–4 in the ‘Aligned’ session, participants took a mandatory 10-minute break. In ‘Rotated’ session that following the break (bottom row of Fig 2), in the ‘Reach to Target’ tasks, the motion of the cursor representing the unseen right hand was rotated about the starting position. The magnitude of the rotation was 30° CW (Fig 1B) for two groups, and 60° CW (Fig 1C) for the other two groups. During the mandatory break, the ‘Instructed’ groups were informed of the rotation and were provided with a strategy to counteract the rotation so that they could still move the cursor in a straight line to their targets. Specifically, they were instructed to visualize the starting position as being at the centre of the clock face. They were told reaching towards a specific number on the clock would result in the cursor heading toward either the next number over (for the 30° rotation), or the second number over (for the 60° rotation) in the clockwise direction. The non-instructed groups were informed that the ‘Reach to Target’ tasks would feel different after the break, but no strategy or details were provided. Both groups were told to keep in mind any strategy they were using to achieve straight reaches, as they would be asked to recall and either use or not use the strategy during some ‘Reach with No Cursor’ trials.

#### Reach to Target tasks

Participants received visual feedback of their hand position via a continuously displayed cursor. This circular cursor, 1 cm in diameter, represented the location of their unseen right thumb. It was green when the cursor moved in the same direction as the unseen hand (Aligned session) and blue when it was misaligned with the direction of the participant’s unseen hand movement (Rotated sessions, Fig 1B and 1C). This misalignment was either a 30° or 60° CW visuomotor perturbation, depending on what group the participant belonged to. After participants placed their hand at the starting position for 300 ms, a target would appear. The target was one of three possible circular disks, 1 cm in diameter, that were situated radially 12 cm away from the starting position at 45, 90 or 135 degrees (Fig 1B and 1C). Participants were told to reach to the target as quickly and accurately as possible. A reach trial would end when the centre of the hand cursor was within 0.5 cm of the target’s centre, while the unseen right hand was held still. When the reach was completed, both the target and the cursor disappeared. Participants then moved their hand back toward the starting position along a robot-constrained straight path, which was generated by a perpendicular resistance force of 2 N/(mm/s) and a viscous damping of 5 N/(mm/s).

The words “Reach to Target” were shown on the screen prior to the start of each set of ‘Reach to Target’ tasks to cue the participant of the next task. There were 45 trials in the first set in the Aligned session and 90 trials in the first set of the Rotated Sessions. These sets were followed by 9 or 30 trials in each top-up set of trials for ‘Aligned’ and ‘Rotated’ sessions respectively (see Fig 2).

#### Reach with No Cursor tasks

These tasks were completed at the end of each block. Participants reached to one of same three radial targets as those in the ‘Reach to Target’ task. However, the cursor that indicated the position of the thumb was not visible and participants were asked to reach to the target without this visual feedback, making these open-loop reaches. When participants believed that they had acquired the target, they held their hand in place for 500 ms, indicating the completion of the reach, and the target disappeared. Participants then moved the robot handle back to the starting position along a robot-constrained path to begin the next trial. As in Werner et al. [5], during the ‘Rotated’ session of the experiment, participants were asked to either employ or not employ any strategy they used during the ‘Reach to Target’ tasks. On-screen instructions prior to each ‘Reach with No Cursor’ task indicated whether they should or should not use their cognitive strategy. Only the participants aware of the rotation and how they were compensating for it were expected to show a clear distinction between reaches employing a strategy and reaches that do not, since awareness of the perturbation is required to dissociate between the two conditions [5]. Each of the three target locations was reached to three times during every ‘Reach with No Cursor’ task for a total of 9 reaches. The order in which ‘Reach with No Cursor’ tasks with and without strategy use were performed were counterbalanced within participants (between blocks, as shown in Fig 2) and between participants.

The words “No Cursor” were shown on the screen prior to the start of each set of ‘Reach with No Cursor’ tasks to cue the participant of the next task. Additionally, during the ‘Rotated’ session, the words “WITH Strategy” or “WITHOUT Strategy” were shown prior to these tasks to inform participants whether to employ their learned strategy.

#### Localization tasks

Localization tasks were used to measure an estimate of the position of the unseen right hand. Similar to the localization tasks used by Izawa et al. [3], and ‘t Hart & Henriques [1], participants were instructed to make outward movements while holding the robot handle with their unseen right hand in a direction of their choosing. The movements were stopped by a force cushion at a distance of 12 cm from the starting position. Participants then moved the robot handle back to the starting position along a robot-constrained path. After moving back to the starting position, they were instructed to indicate the perceived location of their unseen hand at the end of their outward movement using a touchscreen located above the hand (Fig 1A and 1D). Importantly, they received no visual feedback of their right hand while their left hand, used only for localizing their right hand, was entirely visible.

For groups which trained to reach with a 30° rotated cursor during the ‘Rotated’ session, participants moved their unseen hand toward an arc located 12 cm away from the starting position that spanned 60 degrees (similar to the white arc in Fig 1D), centred on the 50°, 90°, or 130° mark in polar coordinates. During each set of localization trials, the arc appeared six times in each of the three possible locations to encourage subjects to move their hand to a large range of locations. The participants indicated their perceived hand location by touching on these same arcs with their visible left hand. For groups which trained with a 60° rotated cursor, participants were instructed to move their unseen hand in a direction of their choosing that fell within a visible V-shaped wedge (Fig 1D), with the tip of the wedge at the starting position and the two ‘arms’ pointing outward. The ‘arms’ of the wedge were 3.5 cm in length and had an angle of 40° between them. This wedge was again used to encourage participants to move to a large range of locations without providing visible targets that may bias touch-screen responses. To keep reach directions consistent between the two groups, the wedges were facing the 50°, 90°, or 130° directions, equivalent to the centres of the arcs experienced by the 30° groups. After completing the movement, an arc appeared 12 cm from the starting position that spanned from 0° to 180° in polar coordinates and participants indicated their felt hand position by touching where they perceived their unseen hand to have crossed that arc. Participants placed their left hand under their chin between taps on the touch screen to avoid confounding contact with the touch screen.

In all ‘Active Localization’ tasks, participants made volitional movements to a location of their choice. After the robot handle was moved 12 cm from the starting position, a force was applied to prevent the participant from reaching further, giving them the sensation of hitting a wall. In passive versions of the localization task, adapted from the task in ‘t Hart & Henriques [1], each participant’s unseen right hand was pulled by the robot manipulandum to various points on the arc. These points on the arc were either identical to the points to which the participants actively reached in the preceding active version of this task or located at the centre point of the arcs (there was no significant difference in localization in either case). Like the active localization task, after hitting a point on the arc, the participants were instructed to return their unseen right hand to the starting position and then indicate with their visible left hand on a touch screen where the right hand intersected with the arc. We find no effects of the positions on the arc which participants’ hands were moved to during the passive version of the task on any of our primary measures. Therefore, to better analyze changes in hand localization, we collapse across these conditions and treat them as one group.

The words “Right Hand: Cross Arc, Left Hand: Touch Cross” or “Robot: Cross Arc, Left Hand: Touch Cross” were shown on the screen prior to the start of each set of localization tasks to indicate to the participant whether the robot would move their unseen right hand or they whether they would do it themselves. These on-screen instructions also reminded the participant to indicate the part of the arc which they thought they crossed during either self-generated or robot-generated movements.

#### Questionnaire

After completing the experiment, participants were asked a series of questions (see ‘supplementary’ document in Open Science Framework project: [26]) similar to that of Benson et al. [16]. The questions were used to quantify how aware participants were of the perturbation and to assess how accurately participants perceived they compensated by using the explicit strategy. Participants are given awareness levels of “None”, “Low” or “High” for receiving Awareness Scores 0, 1, and 3 respectively on the questionnaire.

### Analysis

We aimed to determine the effects of awareness of a visuomotor perturbation during reaching movements on adaptation-related changes in hand localization. To ensure that participants who were instructed or experienced large rotations were in fact aware and used a strategy when adapting to the perturbation, we analyzed reaching movements both during ‘Reach to Target’ tasks and ‘Reach with No Cursor’ tasks. During each reaching movement, both with and without a visible cursor-representation of the hand, angular reach deviations were calculated at the point of maximum hand velocity. The angular deviation was the angular difference between a line from the starting position to the target and a line intersecting both the starting position and the position of a participant’s hand at the point of maximum velocity. When reaching with a rotated cursor representation of the hand, participants had to deviate their reaches by either +30° or +60° (determined by their assigned group) to fully compensate for the rotation, where positive refers to CCW direction. All trials were manually screened to ensure that participants performed as instructed. To make performance during training comparable across the two rotation groups, we also normalize the reach deviations to the size of the experienced rotation.

Estimates of hand location were determined by the angular difference between the endpoint of the unseen right-hand movement, and each participant’s perceived hand position as indicated on the touchscreen. Since participants were free to move their unseen hand in a direction of their choosing, we could not guarantee an even distribution of movements, nor that they moved to specific angles. Therefore, we used kernel smoothing with a 10° wide normal kernel to estimate localization responses at the same hand-movement angles for every participant; 50, 90 and 130 degrees in polar coordinates. These angles were the centre points of guiding arcs and wedges in the ‘Localization’ tasks. The difference between these interpolated hand localization responses in the rotated and aligned session represents the training-induced shift in hand localization. We then used the means of these interpolated values across all three hand-movement angles for all statistical analyses involving hand localization changes. Given that the cursor was rotated CW, localization shifts should also be in the CW, or negative, direction.

First, we analyzed the effects of instruction and rotation size on the process of learning during the first 90 trials of rotated-cursor training. We obtained baseline reach biases for each participant during the last 45 trials of the first “Reach to Target” task in the baseline ‘aligned’ session. To do so, we calculated the average angular error when reaching to each of the three target locations to determine each participant’s baseline reach biases. We then subtracted these individual baseline biases from reach deviations recorded in the ‘rotated’ session to measure angular error. For analysis of reach deviations during training, we grouped the training data into trial sets. For the first 6 trials of rotated training, we define each trial set as 3 reaches, where the participant reaches to each of the three possible targets once. To better represent the asymptote in performance after adaptation, we also used a final trial set of the last 9 trials of training. We then performed a 2x2x3 mixed ANOVA with instruction (*instructed* or *non-instructed*) and rotation size (*30°* or *60°*) as between-subject factors, and trial set (*trial sets 1*, *2*, *or final* during training) as a within-subject factor to examine the effects of instruction and rotation size on performance changes during adaptation. To examine this more closely, we performed 2x2 ANOVAs with instruction and rotation as factors on each of the three analyzed trial sets. This was then repeated for normalized reach deviations. All tests had an alpha level of 0.05 and applied Greenhouse Geisser corrections where necessary.

Next, we assessed the use of explicit strategies during adaptation using the process dissociation procedure (PDP), where participants reached to targets without cursor-feedback. The PDP was adapted from a study by Werner et al. [5] and was used to measure awareness of the perturbation after adaptation. In the PDP, we calculated median reach deviations for each participant when performing reaches while employing any strategy they used during adaptation and when not employing any strategy. First, we determined if adapting to a visuomotor rotation led to changes in reach deviation, that is, reach aftereffects, in ‘Reach with No Cursor’ tasks, for those trials where they were told not to employ a strategy. We ran a 2x2x2 ANOVA on these no cursor reaches with session (*aligned* or *rotated*) as a within-subject factor, and instruction (*instructed* or *not instructed*) and rotation size (*30°* or *60°*) as between-subject factors to confirm that training did lead to reach aftereffects, and to further test if these reach aftereffects changed with instruction or rotation size. After confirming that implicit motor changes (reach aftereffects) occur after adaptation, we subtracted individual biases in reach deviation for each target location during the ‘Reach with No Cursor’ during the aligned session from those in the rotated session. Using these baseline-corrected reach deviations, we ran a 2x2x2 ANOVA on PDP reaches (‘Reach with No Cursor’ tasks during the rotated session) with instruction and rotation size as between-subject factors, and strategy use (*with and without strategy)* as a within-subject factor to examine the effects of instruction and rotation size on performance in the PDP. Awareness of the perturbation would be associated with a significant difference between the reach aftereffects with strategy and without strategy, while lack of awareness would be associated with no difference.

To answer our question whether awareness affects changes in hand localization, we analyzed how afferent and efferent based changes in hand localization due to adaptation were independently affected by instruction and rotation size. First, we wanted to confirm that hand localization shifted with adaptation to a visuomotor rotation, and whether volitional movements in the ‘Active Localization’ tasks lead to additional localization changes. To do so, we compared participants’ estimates of hand position before and after adapting to the perturbations using a 2x2 ANOVA with session (*aligned* or *rotated*) and movement type (*active* or *passive*) as factors within each of the four groups. After confirming there are hand localization changes due to adaptation, we subtracted individual localization biases in the aligned session from those in the rotated session. We then isolated afferent based changes in hand localization (measured in ‘Passive Localization’ tasks where participants did not have access to efferent signals) and efferent based changes in hand localization (the difference between changes in ‘Active Localization’ tasks, where participants did have access to efferent signals, and ‘Passive Localization tasks). This was followed by two 2x2 ANOVAs with instruction (instructed or non-instructed) and rotation size (30° or 60°) as between-subject factors on both sets of data.

To determine if implicit processes such as changes in hand localization and implicit reach aftereffects were related, we explored the relationships between in open-loop reach deviations, and hand localization estimates. We computed Pearson’s correlations between participants’ mean deviations in the ‘Reach with No Cursor’ tasks, i.e. the PDP, and their mean changes in the localization tasks following adaptation. To examine if awareness of the perturbation on an individual level affected hand localization changes, we also computed Spearman’s correlations comparing either afferent or efferent-based shifts in hand localization and Awareness Scores on post-experiment questionnaires. Finally, to determine if Awareness Scores from post-experiment questionnaires are reliable in measuring awareness of the perturbation, we perform an ordered logistic regression to see if either providing instruction or experiencing a larger perturbation increases the odds of getting higher Awareness Scores.

Python version 3.6 [27] was used for data preprocessing [28,29] and figures [30,31]. Statistical analysis was conducted in R version 3.4.4 [32,33].

## Results

### Manipulating and measuring awareness of the perturbation during adaptation

We set out to determine if visuomotor adaptation-related changes in hand localization, both due to proprioceptive recalibration and the updating of predicted consequences, were modified by awareness of the perturbation. To test this, we measured hand localization after participants adapted to 30° or 60° visuomotor rotations and compared them to their hand localization estimates prior to adaptation. Crucially, some participants also received instructions on countering the rotation, making them aware of the perturbation. We also conducted a process dissociation procedure (PDP) to ensure that larger perturbations and instructions resulted in increased awareness of the perturbation.

**Fig 3:**
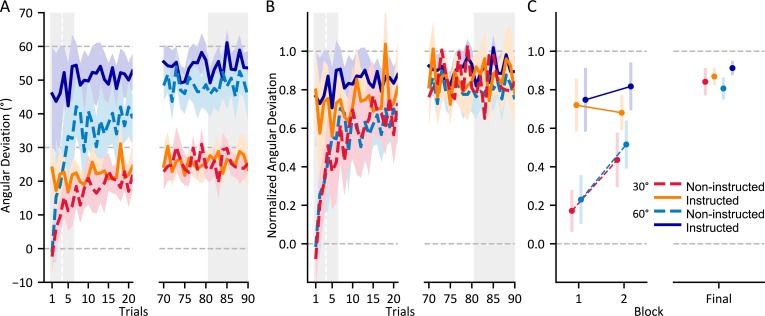
Performance of all groups during adaptation. A) Reach deviations in the direction countering the perturbation for the first and last 20 trials of training. The indicator lines at 30 and 60 degrees demonstrates the reach deviation required to fully counter the perturbation for groups that experience a 30 and 60-degree rotation respectively. B) Results in A normalized with respect to rotation size. The indicator line at 1.0 demonstrates full compensation of the perturbation. C) Normalized mean reach deviations for the first two three-trial sets of training and the final nine trials of training used in the ANOVAs, as indicated by the grey area in A and B. Shaded areas and error bars are 95% confidence intervals.

As shown in Fig 3A, all groups showed trial set by trial set learning (main effect of trial set; F_(2)_ = 56.900, p < 0.001, generalized eta squared (η^2^_*G*_) = 0.256). When exposed to a visuomotor rotation, participants in all groups learned to deviate their reaches to counter ~87% of the rotation by the end of 90 training trials. To examine the effects of instruction and perturbation size on the extent of our participants’ adaptation, we analyzed the first trial set of training where we expect to see the largest effects. In the first trial set, both instruction (main effect; F_(1,81)_ = 47.754, p < .001, η^2^_*G*_ = .371) and rotation size (main effect; F_(1,81)_ = 21.467, p < .001, η^2^_*G*_ = .210) significantly affected hand deviation (Fig 3A). However, when reach deviations were normalized relative to the rotation size (Fig 3B and 3C), we find that only instruction (main effect; F_(1,81)_ = 62.905, p < .001, η^2^_*G*_ = .437), and not the rotation size (main effect; F_(1,81)_ = 0.409, p = .524, η^2^_*G*_ .005) lead to greater initial compensation. As illustrated in Fig 3B, the two instructed groups adapted similarly and the two non-instructed groups adapted similarly over the 90 training trials. The difference in reach deviation due to instruction persisted throughout the second trial set of training (main effect; F_(1,81)_ = 21.518, p < .001, η^2^_*G*_ = .209) and even until the end of the training task (main effect; F_(1,81)_ = 6.899, p = .010, η^2^_*G*_ = .078). In short, as seen in Fig 3, instruction but not rotation size led to more substantial compensation during training.

**Fig 4:**
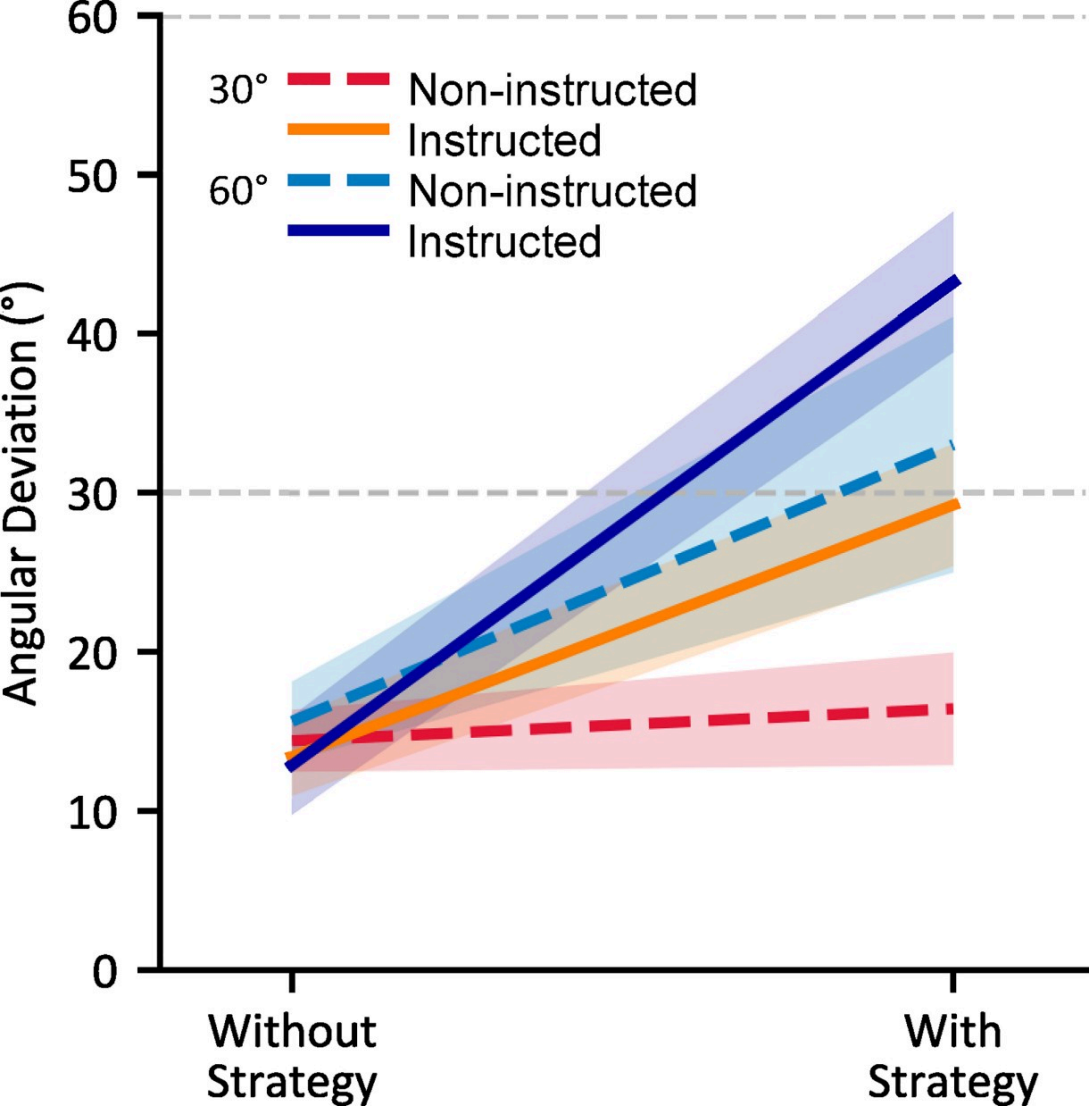
Changes in no-cursor reaches following training. Mean group deviations in movement direction, while suppressing (left side) or employing (right side) any strategies employed during adaptation. Shaded areas and error bars are 95% confidence intervals.

Next, we confirmed that adapting to a rotated cursor lead to significant reach aftereffects (continued reach deviations when reaching without a visual cursor representation of the hand). As seen in Fig 4, reach aftereffects arise in all groups when they are not employing any learned strategies, but the groups have different reach aftereffects in open-loop reaching tasks where they do employ a strategy. Hand-deviations in the ‘Reach with No Cursor’ tasks where participants were not told to use the explicit strategy used during training were deviated 14.0° on average in the direction countering the visuomotor rotation in the rotated session when compared to the aligned session (main effect of session; F_(1,81)_ = 518.821, p < .001, η^2^_*G*_ = .683). These implicit aftereffects of adaptation were similar in size for all four groups (Fig 4A: Without Strategy), independent of instruction and even the size of the rotation (no interactions between session and either instruction or rotation size). This suggest that implicit aftereffects are not suppressible and are rigid in their magnitude. This is despite differences in the size of the experienced perturbation, and even awareness of the perturbation during adaptation.

To test our prediction that both strategies and large perturbation sizes would result in awareness of the perturbation during adaptation, we examined the ‘Reach with No Cursor’ trials where participants used any explicit strategies they employed during adaptation (Fig 4A: “With Strategy”) and compare them with trials where participants do not use any strategy. We tested whether participants were able to evoke a strategy when cued during the PDP, i.e., to further deviate their hand in reaches with strategy use when compared to reaches without strategy use. Reach deviations with a strategy, on average, were larger than those produced when asked to exclude any conscious strategy (main effect of strategy use; F_(1, 81)_ = 119.002, p < .001, η^2^_G *=*_ .460). Specifically, being instructed (instruction * strategy use interaction; F_(1, 81)_ = 19.817, p < .001, η^2^_*G*_ = .124) and experiencing a large perturbation (size * strategy use interaction; F_(1, 81)_ = 24.754, p < .001, η^2^_*G*_ = .151) led to greater reach deviations when asked to use strategy than when asked not to. This suggest that training with a larger rotation even without instruction is sufficient to develop awareness of the nature of the perturbation. As illustrated in Fig 4, only the non-instructed 30° group (red) did not show significantly different angular deviations when including a strategy (M = 16.437, SD = 7.578) as compared to not including one (M = 14.425, SD = 4.166; t_29.5_ = -1.041, p = 0.307). However, although participants who experience a large perturbation developed strategies to counter it, neither group which adapted to a 60° rotation could employ a strategy to counter the full magnitude of the rotation (the non-instructed 60° group countered 70% and instructed 60° group countered 77% of the rotation when asked to use strategy). Our results suggest that during adaptation to visuomotor rotations, either receiving instruction or training with a large perturbation can lead to awareness of the perturbation, as well as a strategy for how to counter it. However, there may be factors at play when countering large perturbations that cannot be captured via implicit learning or explicit strategy use.

### The effects of awareness during adaptation on hand localization

**Fig 5:**
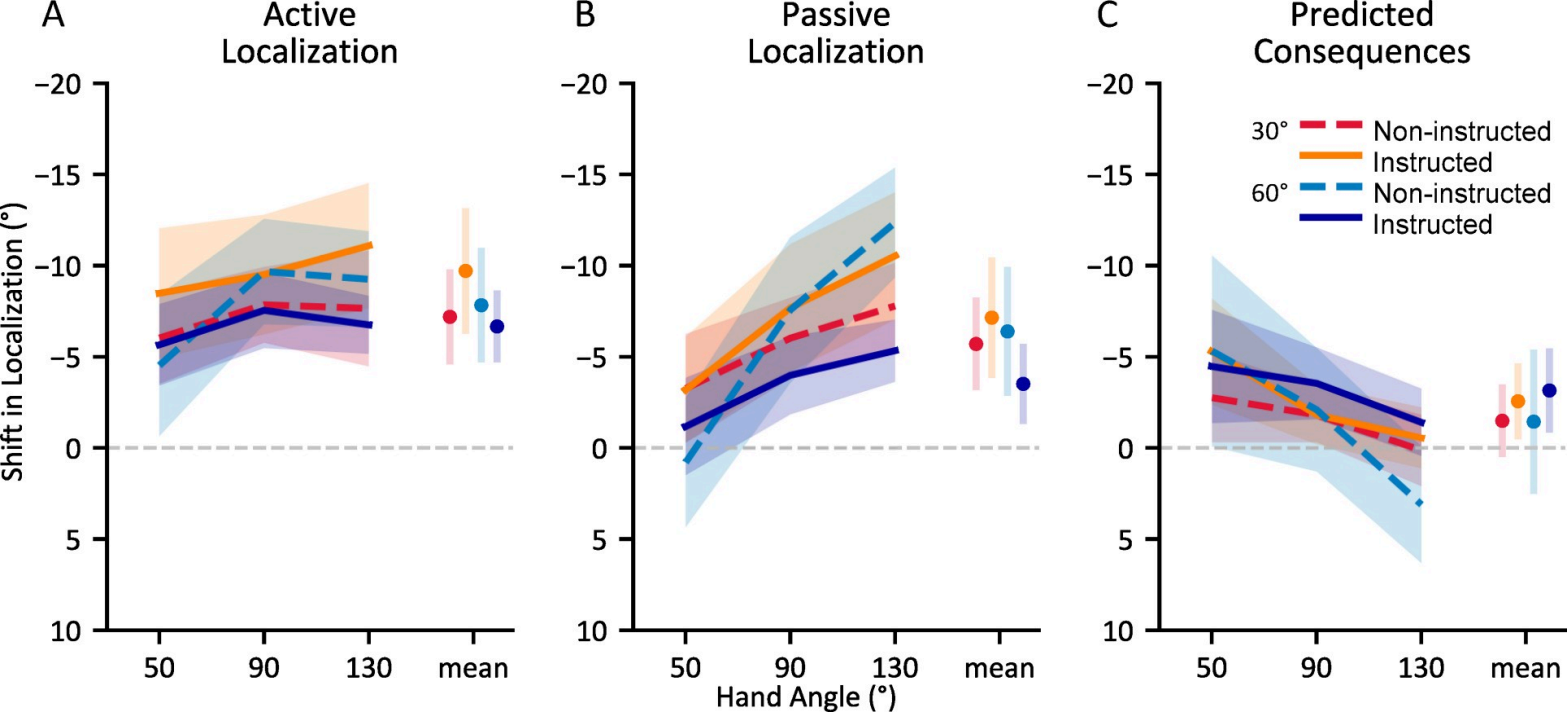
Changes in localization of the unseen, adapted hand following visuomotor adaptation. A) After self-generated movements; Active Localization B) After robot-generated movements; Passive Localization C) Difference between Active and Passive Localization, intended to capture updates in predicted sensory consequences (efferent based estimates). Shaded areas and error bars are 95% confidence intervals.

Having confirmed that instruction and large perturbation sizes lead to increased awareness of the nature of the perturbation during adaptation, we examine the effects of differences in awareness on changes in estimates of hand position (Fig 5). Specifically, we probe both afferent (via available sensory information) and efferent (via an efference copy of produced motor commands) based changes in localization of the hand following adaptation. To isolate these changes, we use one localization task in which participants have access to both efferent and afferent based signals of hand location (the ‘Active Localization’ task; Fig 5A) and another in which participants only have access to an afferent based signals of hand location (the ‘Passive Localization’ task; Fig 5B). By comparing the two tasks, we attempt to probe changes in hand localization based only on efferent based information (Fig 5C).

All groups showed a change in hand localization following adaptation (main effect of session (aligned vs rotated); F_(1, 84)_ = 172.036, p < .001, η^2^_*G*_ = .177). These changes were modulated by the type of movement of the hand being localized (session * movement type interaction; F_(1, 84)_ = 30.048, p < .001, η^2^_*G*_ = .006), suggesting a further shift in efferent-based perceived hand position when the hand was actively moved by the participant. A majority of the observed shift, 5.6°, was afferent-based, present in the ‘Passive Localization’ task. Neither instruction nor rotation size affected this proprioceptive recalibration of hand localization (F_(1, 81)_ = 0.437, p = .510, η^2^_*G*_ = .005) and F_(1, 81)_ = 1.851, p = .177, η^2^_*G*_ = .022 respectively). That is, proprioceptive recalibration was not modulated by instruction, and saturated at around 5.6°, no matter how large the perturbation or adaptation. However, there was a weak interaction (η^2^_*G*_ = .047) between instruction and rotation size on afferent-based changes in hand localization (F_(1, 81)_ = 3.973, p = .050). Although instructed participants who experienced a 60° rotation did show slightly diminished afferent-based changes in hand localization, this effect was not significant after post-hoc adjustments (Tukey’s HSD: all p > 0.05).

When we isolate efferent-based shifts in hand localization, we find 2.2° of additional shift (about 38% larger) compared to the afferent based changes present in ‘Passive Localization’ tasks (Fig 5: panel A vs B, illustrated in panel C). These additional shifts can be attributed to updating of efferent based estimates of hand position. However, as for afferent based changes, neither instruction (F_(1, 81)_ = 2.974, p = .09, η^2^_*G*_ = .035) nor rotation size (F_(1, 81)_ = 0.113, p = .694, η^2^_*G*_ = .002) significantly modulated these changes. Thus, our results show that neither instruction nor rotation size have measurable effects on changes in either efferent or afferent based hand localization (Fig 5).

**Fig 6:**
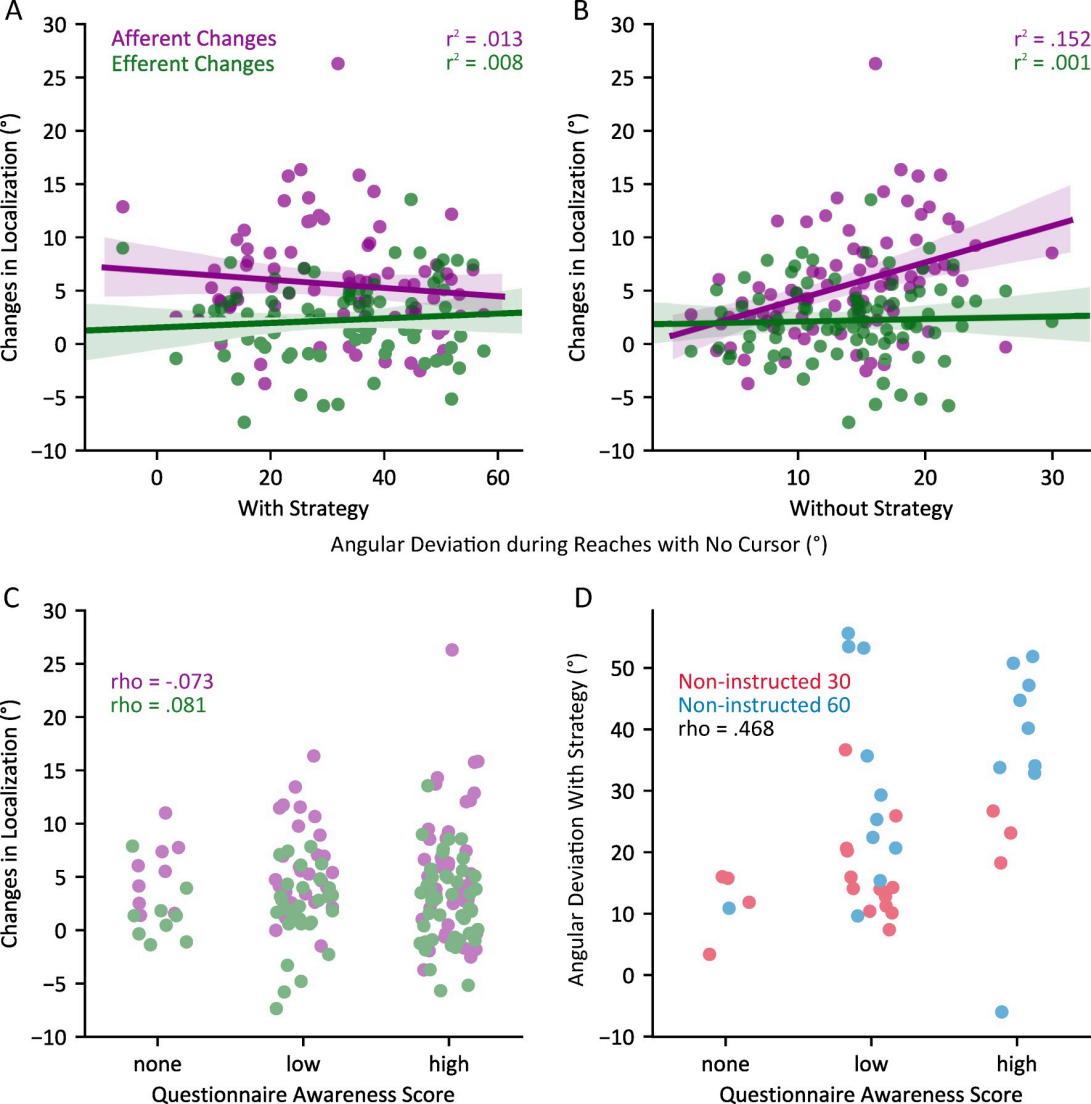
Relationships between changes in hand localization, angular deviations during reaches with no cursor, and awareness of the perturbation. Relationships between afferent and efferent based changes in hand localization following adaptation and angular deviations without a cursor representation of the reaching hand while employing (A) and supressing (B) any explicit strategies used during adaptation. C) Relationships between changes in hand localization following adaptation and Awareness Scores in post-experiment questionnaires. D) Relationships between angular deviations without a cursor representation of the reaching hand while employing a strategy and Awareness Scores in post-experiment questionnaires in the non-instructed groups.

We find that afferent and efferent-based changes in hand localization were not correlated with participants’ hand deviations during ‘Reach with No Cursor’ trials in which they use an explicit strategy (Fig 6A: r = -.114, p = .300 and r = .088, p = .426 respectively). However, as illustrated in Fig 6B, afferent based localization changes were significantly correlated with reach deviations during ‘Reach with No Cursor’ trials where participants did not use an explicit strategy, i.e., implicit motor changes or reach aftereffects due to adaptation (r = .390, p < .001), although efferent-based localization changes were not (r = .036, p = .747). Our findings suggest that, for the most part, efferent and afferent changes in hand localization, and motor changes due to adaptation are separate processes, but there may be similar mechanisms between afferent-based changes in localization and implicit motor aftereffects of adaptation. That is, proprioceptive recalibration partly predicts implicit motor changes.

To further test whether awareness of the perturbation during adaptation affects changes in felt hand position at an individual level, we also test whether Awareness Scores from self-reported questionnaires [26] are correlated with their changes in hand localization. Consistent with our analysis above, Awareness Scores were not correlated with either afferent based (*ρ = -*.073, p *=* .503) or efferent based (*ρ* = .081, p *=* .461) changes in hand localization (Fig 6C). Self-reported questionnaire responses were poor representations of awareness levels when compared to behavioural measures such as the PDP [5]. We added scores to our questionnaires to improve the efficacy of self-reported questionnaires. When assigning an Awareness Score (none, low, or high) to the responses on the questionnaire used in Benson et al. [16], we find that receiving instruction increased the odds of having a higher awareness score (proportional odds ratio (OR): 5.086, p < 0.001), but adapting to a larger rotation size did not (OR: 1.685, p = 0.242).

Both Awareness Scores on post-experiment questionnaires and PDP measures show we were successful at evoking explicit awareness of a perturbation during adaptation, both by instructing participants and by having them adapt to large perturbations. Our results taken together indicate that changes in hand localization due to adaptation, both when relying on afferent-based and efferent-based information are largely implicit processes. High awareness levels in various awareness measures, i.e., participant questionnaires and process dissociation procedures, do not result in any significant changes in the shifts in localization of the adapted hand, with possibly a smaller change in proprioceptive recalibration for the most aware group (given both a large perturbation and instruction). That is, implicit components of motor adaptation, including the localization changes, are rigid, found in all groups, independent of cognitive factors.

## Discussion

We test whether explicit motor learning due to awareness of a perturbation during adaptation, brought about by either instruction or by experiencing a large 60° visuomotor rotation, can modulate changes in the estimation of hand position. We find that adapting to a visuomotor rotation leads to significant changes in hand localization, which are both afferent based, informed by sensory information from the effector, and efferent based, informed by a copy of a motor command during a movement. We find that both instructions and large rotations affect measures of explicit learning, but they do not impact changes in estimates of hand position. As we will discuss below, our findings have implications for the processes involved in both proprioceptive recalibration and updating of predicted sensory consequences.

### Explicit learning and awareness of the perturbation

Contributions of explicit and implicit components to motor learning have been measured in various ways. This includes using aiming tasks where participants indicate their employed aiming strategies prior to reaching with the rotated cursor [6,15,17,34,35], or using aftereffects of reach adaptation or post-experiment questionnaires for instructed and non-instructed groups [5,7,16]. Where measured with an aiming task, explicit learning appears to dominate in early sessions of adaptation, when errors are large and salient [6,8,9,15]. Our results are consistent with this early contribution of explicit learning; the benefits of instruction were largest in early trials of adaptation (effect size for the first 3 trials, η^2^_*G*_ = 0.437), although they persist up to 90 trials into training (η^2^_*G*_ = 0.067). Participants who experienced a larger perturbation but did not receive instructions adapted at faster absolute rates, but these changes in reach deviations were proportional to the rotation size. As adaptation progresses, the contribution of explicit learning decreases as implicit aspects of learning slowly increase and begin to dominate the adaptation process [15]. Although we do not measure explicit components of learning during training, like Benson et al. [16], Werner et al. [5] and Neville & Cressman [7], we see evidence for potential benefits of having well-informed cognitive strategies during early adaptation. That is, our results suggest instruction on the nature of a perturbation may lead to increased explicit components of learning even when perturbations are not salient.

Although performance as a whole is similar between people who experience the two rotation sizes when normalized, measures of explicit components of adaptation, including awareness of the perturbation following adaptation, have been repeatedly shown to differ with the size of the perturbation [5–7,17,18]. We measured the consequences of these explicit components of learning following reach training using the process dissociation procedure (PDP: Fig 4). When non-instructed participants reached with no visual feedback of the hand, people who trained with a 60° visuomotor perturbation were able to evoke a strategy when asked, but those trained on the 30° rotation were not. This suggests that the larger rotation was sufficient to elicit explicit learning. These PDP results are consistent with similar measures of explicit learning studies using large perturbations of 75° [18], 60° [5,7] and even 40° [7]. Likewise, these same studies show no such evidence for evoked strategy after adapting to small perturbations, such as 20° [5,7] or 40° [5]. This suggests that rotation sizes greater than 40° are more likely to elicit persistent awareness while smaller rotations either do not elicit awareness, or what is elicited is too transient to last post-adaptation. Conversely, when explicit components of learning were solicited during training, via aiming tasks, even rotation sizes as small as 15° are sufficient for the development of a strategy which persists throughout training [6]. Nonetheless, this explicit contribution was proportionally smaller, and indeed, the percentage of the explicit contribution to overall learning grew with larger rotations. Although such immediate measures of explicit learning are informative about its expression during adaptation, the task of choosing an aiming direction prior to reaching may lead to additional explicit learning [5,15]. By using the PDP, we avoided overexpression of explicit components in non-instructed groups. Using this method, we show that non-instructed participants whom experience a 30° rotation are not aware of the perturbation whereas those that experience a 60° perturbation are.

A reliable method of evoking awareness of the perturbation during training is simply informing the participants of the perturbation and how to counter it. When we provided such instruction to participants, we observed increased strategy use in people that adapted to both large and small rotations (Fig 4B: instructed groups). Indeed, for a small rotation of 30°, instructed participants on average evoke a complete strategy, showing reach deviations in the absence of the cursor that matched the perturbation. For the larger 60° rotation, reach deviations produced when evoking the strategy are not nearly as complete, although this is generally the case seen in other studies (e.g., Hegele & Heuer [18]). Like in our study, Werner et al. [5] and Neville & Cressman [7] find that providing instruction increased the ability to evoke an explicit strategy when cued during a PDP task, regardless of the size of the experienced perturbation. As with these previous studies, our PDP measures show that instructing participants and having them experience large perturbation sizes each lead to awareness of the perturbation, with slightly greater reach deviations for the group that had both (instructed 60°); although this does not reach statistical reliability. Overall, by using post-training measures of perturbation awareness, we were successful in evoking awareness in some groups (both instructed groups and the non-instructed 60° group) and not others (non-instructed 30° group), allowing us to cleanly study its effects on other consequences of motor adaptation.

Implicit components of adaptation are likely independent of explicit components, differing in both the time course of their development during training [8,15] and their development during the aging process [17]. However, they may share common neurophysiology, as patients with cerebellar ataxia show deficits in both implicit and explicit aspects of reach adaptation [36]. We find that reach-aftereffects made without a strategy, which should only reflect implicit adaptation, were consistent in their magnitude regardless of instruction or the size of the perturbation. As in our study, Bond and Taylor [6], as well as Neville and Cressman [7], find that implicit aftereffects are of similar size independent of the size of the visuomotor rotation. However, others have shown that implicit aftereffects can be proportional to the rotation size, but these cases tend to either gradually introduce the perturbation [20,37] or include multiple rotation sizes during training [5,38]. Introducing multiple rotation sizes or introducing a perturbation gradually may increase implicit components of learning by exposing the participant to smaller required adjustments. We can only speculate at the mechanisms as this is beyond the scope of this study. While we find no effects of instruction on implicit reach aftereffects, Neville & Cressman [7] find that providing instruction can lead to smaller implicit aftereffects of learning, measured by the PDP, although again, they did not eliminate these persistent reach deviations. Overall, as expected, the implicit contribution to overall learning is far less sensitive to instruction, and even perturbation size, than the explicit contribution.

### Hand localization

The roles of instruction, strategy and explicit learning on resulting changes in hand localization have not been explored. While it has been shown that motor adaptation leads to both afferent and efferent based changes in hand localization [1,2,4], it is not clear if both changes are entirely implicit, or if either one can be modulated by explicit components of learning. Awareness of the perturbation may lead to experienced errors being assigned to an external source rather than one that is internal, which has been shown to affect adaptation [39–41], and thus should not lead to changes in body-based estimates. However, regardless of whether participants are given instructions prior to adaptation, and regardless of whether they exhibit awareness of the perturbation following training, participants did not show any effects of instruction or rotation size on changes in either proprioception or efferent-based hand localization. At most, we found a near-significant interaction of instruction and rotation size for proprioceptive recalibration, likely due to the smaller proprioceptive shift for the instructed 60 group compared to non-instructed 60° group. But overall, we find no evidence that localization changes are affected by awareness of the perturbation, suggesting they are largely implicit.

Moreover, the size of these shifts in localization did not vary with the size of rotation. This is in contrast to an earlier study from our lab [20] where gradually introducing the cursor rotation and subsequently further increasing its rotation may have led to proportionally larger changes in hand estimates (as well as implicit reach aftereffects, as mentioned above) after sufficiently training with each of the three final rotations; roughly accounting for 20% of rotations of 30, 50 and 70 degrees. Gradually introducing the different rotations would have made the errors consistently small across trials and perturbation sizes. This may have led to both motor and sensory recalibration that did not saturate at the same magnitude as it did in the current study, where the perturbation was salient and the errors, at least originally, large. It is possible that the changes in localization and reach aftereffects are capped by the explicit learning evoked by the larger rotation and otherwise have the potential to be different across the two rotations. The extent that errors may have been intrinsically attributed to the body may have dictated the extent to which both estimates and open-loop movements of the hand could be altered. While gradually introducing the perturbation, such that errors always remained small, which may lead to a larger proportion of errors being attributed to intrinsic properties than when experiencing an abruptly introduced rotation. Further studies are needed to explore the role of error sensitivity on both explicit and implicit learning, as well as the recalibration of hand estimates.

Although explicit components of learning may lower the ceiling for changes in hand localization, our study suggests that, like the motor aftereffects of adaptation, changes in perceived hand location are also not completely suppressible. Changes in afferent-based hand localization, i.e., proprioceptive recalibration, have however been shown to be separate from motor changes; with different time courses [21] and generalization patterns [42]. Likewise, as in this study, these changes in proprioceptive estimates are much smaller than the reach aftereffects. However, we do find a modest relationship between implicit motor and proprioceptive recalibration in this large sample size (Fig 6B), suggesting that part of these reach aftereffects, observed when participants do not employ any conscious strategy, may be in part driven by changes in proprioception [43]. Hand localization, and its shifts due to motor adaptation are informed both by afferent signals, such as proprioception, and efferent signals, like the motor commands to generate volitional movements, the latter of which may be altered by the changed internal forward model after adaptation [10,11]. Synofzik et al. [25] and Izawa et al. [3] have found significant changes in estimates of hand position, using an active localization task, following adaptation [3,25,44]. As replicated in our study, when a passive version of the localization task was used to isolate afferent and efferent-based changes in hand localization, more than half of a 10° shift in hand localization could be attributed to changes in afferent-based localization [1]. This is consistent with the additional results of Synofzik et al. [25] and Izawa et al. [3], where they found that the learning-induced shifts in active hand localization were present, although reduced, in patients with cerebellar damages, and our own results, where cerebellar patients do show proprioceptive recalibration [45]. This suggest that indeed the cerebellum may be contributing to predicting the sensory consequences of movements but not proprioceptive estimates of hand position [45,46]. We also isolated these components and observed how they interact with awareness, and indirectly source attribution of errors, during adaptation. When we isolated these efferent-based changes in estimates of hand position, they, much like afferent-based changes, were also not modulated by awareness of the perturbation. Our finding suggest that these efferent based changes too are implicit in nature.

### Measuring awareness

When determining awareness, relying solely on questionnaires may lead to its underestimation as verbal and motor responses are significantly different retrieval contexts [47]. Furthermore, questionnaire responses on higher order cognitive processes are held in low confidence, as responses may be effected by multiple factors including the saliency of stimuli related to the response, levels of attempted introspection and even conscious access to cognitive components of prior performance [48,49]. Thus, we used a variation of a process dissociation procedure (PDP), adapted by Werner et al. [5], to objectively measure explicit learning following adaptation. Werner et al. [5] found that PDP results were informative, related to performance during adaptation and during catch trials which measured implicit adaptation, whereas binary questionnaire results were not. Although the PDP may be a more principled method of measuring the results of awareness of the perturbation, we find that adding a scoring system to the questionnaire [26] used by Benson et al. [16], where different degrees of awareness are accounted for, can provide insights on participant awareness (Fig 6A). Non-instructed participants’ scores on the modified questionnaire correlate moderately with open-loop reaches when participants employ a strategy (the main distinguishing variable of the PDP; Fig 6D, *ρ =* .468, p = .002), while no such relationship arises when the outcome of the questionnaire is dichotomous [5]. In an ambiguous situation, as in the non-instructed 60° group where some participants develop an explicit strategy, questionnaire responses, although non-tractable, may be an asset when performance in the PDP is not measurable.

## Conclusion

Having instructions on the nature of a visuomotor perturbation before experiencing it, as well as experiencing a large perturbation, lead to the development or use of an explicit strategy during adaptation. Even when adaptation involves these explicit processes, implicit aftereffects of adaptation, in the form of continued reach deviations in the absence of a perturbation, are present. What’s more, the magnitude of these aftereffects is independent of the size of the perturbation, and whether participants are given a strategy to counter the perturbation. Adapting to a visuomotor rotation also leads to changes in estimates of unseen hand position. Hand-localization estimates, which are based both on afferent information, from sensory inputs such as proprioception, and efferent information, derived from efference copies of motor commands, both significantly shift to more align with visual input during adaptation. These changes in hand localization are not modulated by awareness of a perturbation or the use of a strategy during adaptation. Our findings support the notion that both proprioceptive recalibration and efferent based changes in hand localization are irrepressible and largely implicit in nature. Additionally, like other implicit components of motor learning, these sources of hand-position estimates develop independently from explicit components, and possibly, even from each other.
